# Supplementary material for: Hierarchical tensile structures with ultralow mechanical dissipation
Source: Nat Commun. 2022 Jun 2;13:3097. doi: 10.1038/s41467-022-30586-z (PMC9163184; doi:10.1038/s41467-022-30586-z)
Supplement: Supplementary file 1 — Supplementary Information [file 41467_2022_30586_MOESM1_ESM.pdf]

Supplementary Information for: "Hierarchical tensile structures with ultralow mechanical dissipation"

M. J. Bereyhi[1,*], A. Beccari[1,*], R. Groth[1,*], S. A. Fedorov[1,*], A. Arabmoheghi[1], T. J. Kippenberg[2], & N. J. Engelsen[3]

[1]*Institute of Physics, Swiss Federal Institute of Technology Lausanne (EPFL), 1015 Lausanne, Switzerland*

∗*These authors contributed to this work equally.*

[2]*tobias.kippenberg@epfl.ch*

[3]*nils.engelsen@epfl.ch*

1 Theoretical quality factors of high-stress resonators

The intrinsic loss-limited quality factors of flexural modes in high aspect ratio resonators subjected to static tension are controlled by dissipation dilution [1–3]. The quality factor, Q, of a mode can be theoretically calculated using the formula [4]

$$Q = D_Q \times Q_{\text{int}}, \tag{1}$$

where Q_{int} is the intrinsic quality factor and D_Q is the dilution coefficient. For the Si_3N_4 films used in our work, Q_{int} was experimentally characterized as described in the following section. The dilution factor, D_Q, is equal to the ratio of geometrically nonlinear (tension) and linear (bending and torsional) elastic energies [4]. It depends on the film pre-strain, resonator geometry, and the

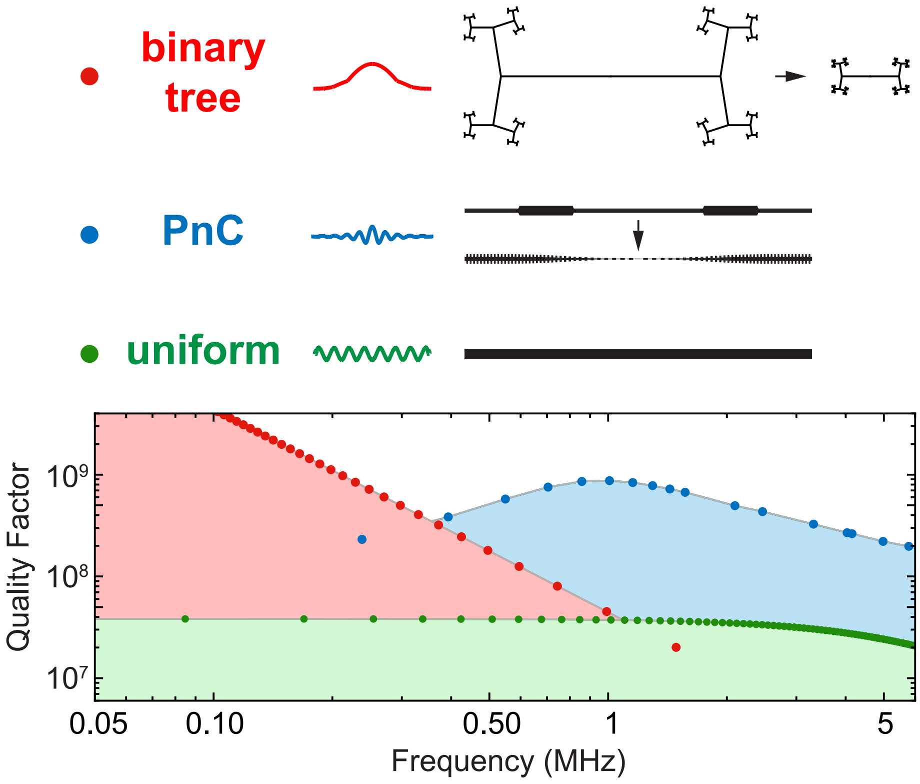

Figure 1: Detailed quality factor landscape of different resonator designs. Points show theoretically computed quality of high-stress Si_3N_3 resonators. Red points: fundamental modes of binary tree resonators; blue: localized modes in phononic crystal beams with strain engineering; green: modes of a uniform beam. The legend shows typical mode shapes and evolutions of resonator geometries from low to high frequencies where applicable.

vibrational mode order. We find D_Q using either finite element simulations, in which our resonators are modeled as plates and the ratios of the energies are evaluated as described in the SI of Ref. [2], or quasi one-dimensional models from [4,5] whenever they agree well with full simulations.

Analytically, the dilution factor can be expressed as

$$D_Q = \frac{1}{\alpha\lambda + \beta\lambda^2}, \tag{2}$$

where α and β are the boundary and the distributed loss coefficients, respectively, and λ is the stress parameter,

$$\lambda = \frac{h}{l}\sqrt{\frac{\sigma}{12E}}. \tag{3}$$

Here h is the film thickness, l is the resonator length, σ is the static stress and E is the Young's modulus. The precise definitions of σ and l depend on the resonator type: whether it is a beam, a membrane or a binary tree. In resonators with high dilution (for some devices in our work D_Q is as high as 2×10^5), the stress parameter is much smaller than one and the boundary term naturally dominates the denominator of Eq. 2. This means that the dissipation is dominated by the internal friction in the material in the immediate vicinity of the clamps even if the physical properties are uniform over the resonator surface. Vibrational modes with negligible boundary loss coefficients (i.e. with $\alpha \to 0$), are called soft-clamped [6].

In Fig. 1, we present a comparison of theoretically calculated diluted quality factors achievable with different resonator types. Since dissipation dilution increases monotonously with increasing aspect ratio, we impose a length limit of $3\,\mathrm{mm}$ and assume a fixed film thickness of $20\,\mathrm{nm}$ in order to make a meaningful comparison between different geometries. We use the following ma-

terial parameters: density of $\rho = 3100\,\mathrm{kg/m^3}$, Young's modulus of $E = 250\,\mathrm{GPa}$, Poisson's ratio of $\nu = 0.23$, deposition stress of $\sigma_{\mathrm{dep}} = 1.03\,\mathrm{GPa}$, and intrinsic quality factor of $Q_{\mathrm{int}} = 2500$.

Green points in Fig. 1 correspond to the modes of a $3\,\mathrm{mm}$-long uniform beam with rectangular cross section, whose dilution factors are given by [1,7]

$$D_Q = \frac{1}{2\lambda + (\pi n \lambda)^2}, \tag{4}$$

where n is the mode order. This resonator type is one of the most widely experimentally studied to date. Modes of beams shorter than $3\,\mathrm{mm}$ would fall into the green shaded area.

Blue points correspond to soft-clamped modes in $3\,\mathrm{mm}$-long phononic crystal beams incorporating strain engineering techniques [8]. Here each point corresponds to a separate beam design, for which the unit cell number (and hence the localized mode order) was fixed and the phononic defect and tapering parameters were chosen to maximize the quality factor. All other modes of these structures, soft-clamped modes at sub-optimum parameters, and all modes of smaller beams of this type have Qs in the blue shaded area, or in the other shaded areas which are yet below.

Red points correspond to fundamental modes of binary tree beams with $N = 5$ generations of branchings, found using the model from ref. [5]. The geometric parameters ($r_l = 0.45$, $\theta = 81°$) have been optimized to maximize the Q at a fixed length. l_0 was swept between $100\,\mathrm{\mu m}$ and $3\,\mathrm{mm}$ in the plot. The fundamental modes of sub-optimum designs would fall into the red shaded area or below. Fig. 1g in the main text shows lines that are interpolated between the points shown in Fig. 1.

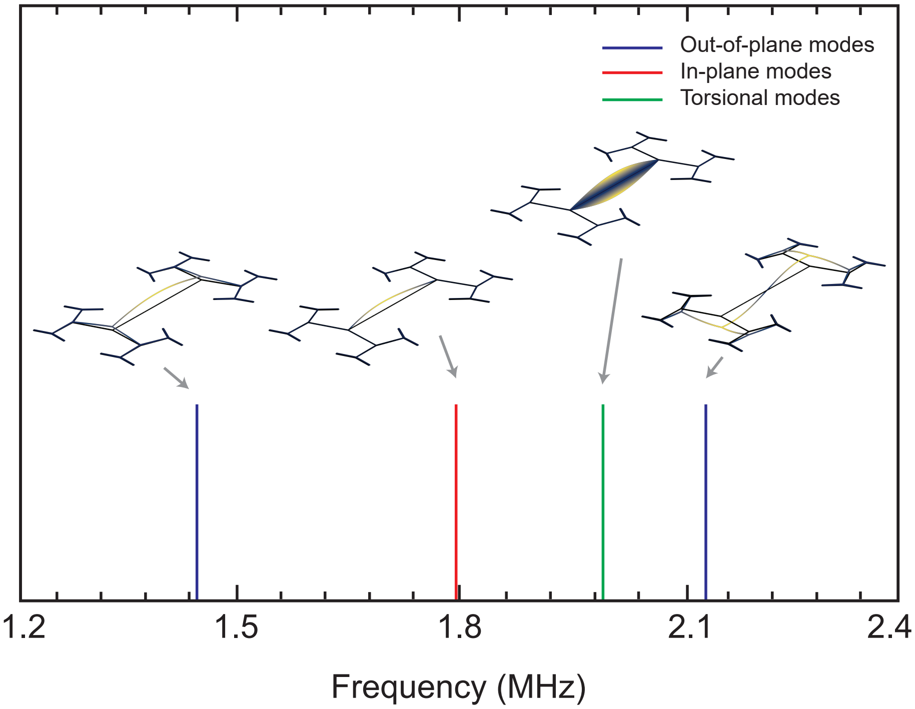

Figure 2: Lowest-order modes (out-of-plane, in-plane and torsional) of a Si_3N_4 binary tree resonator with $l_0 = 70\,\mu\mathrm{m}$, $r_l = 0.45$, $\theta = 78^o$, $N = 3$, $w_0 = 1\,\mu\mathrm{m}$ and $20\,\mathrm{nm}$ thickness.

2 Mode separation in binary tree beams

We simulate the modes surrounding the fundamental out-of-plane mode to quantitatively show the frequency separation between this mode and the in-plane and torsional modes closest in frequency (Fig. 2). Compared to elastic strain engineering nanobeams presented in [8], for a similar frequency range ($\sim 1\,\mathrm{MHz}$) the mode separation between the out-of-plane mode and the lowest-order in-plane ($350\,\mathrm{kHz}$) and torsional ($510\,\mathrm{kHz}$) modes are larger by one order of magnitude.

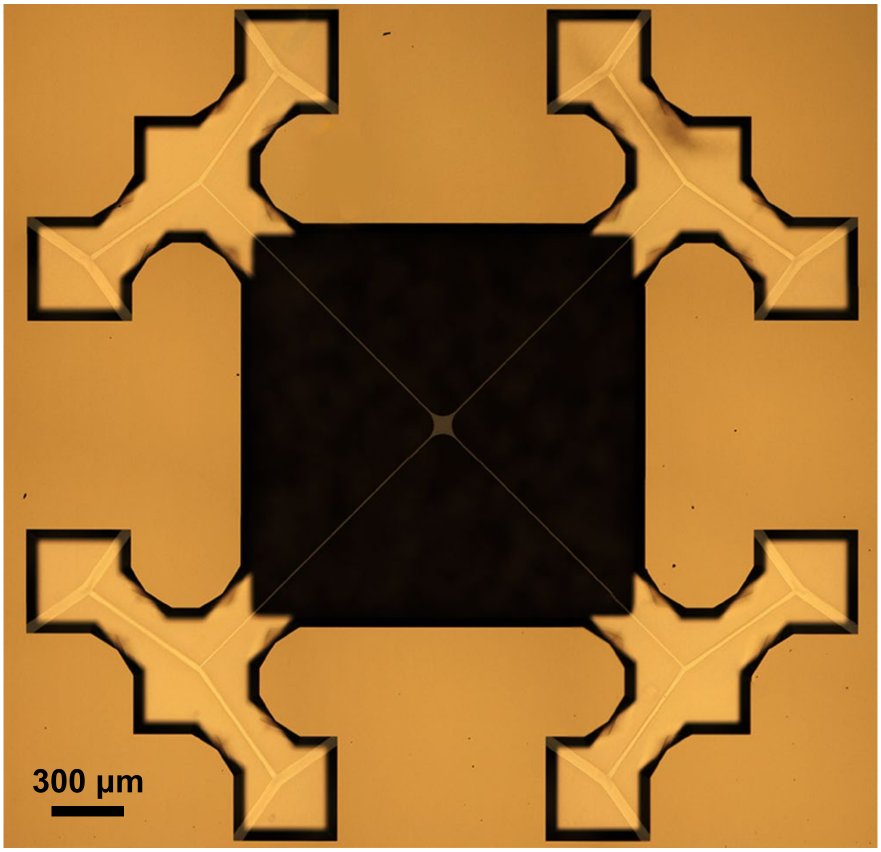

Figure 3: Optical micrograph of a self-similar trampoline membrane suspended over an optical window that perforates the silicon chip. The membrane thickness is $20\,\mathrm{nm}$, while the chip is about $200\,\mu\mathrm{m}$-thick.

3 Integration of a backside window with self-similar trampolines

In cavity optomechanics experiments, membrane devices are commonly embedded in a Fabry-Pérot cavity to realize a membrane-in-the-middle configuration: this requires optical access to the membrane pad with a transmission window etched through the chip. The device in Fig. 4b of the main text is suspended over such an optical window, but for the self-similar trampoline in Fig. 4a, the window was not fabricated. We omitted the backside window in order to improve the survival yield of these fragile devices after the release. Though we did not attempt it with this specific design, fabrication of a backside window is possible with the process described in the previous section.

We show in Fig. 3 a self-similar trampoline device with a backside window opened through the chip thickness. As mentioned above, this requires splitting the KOH undercut in two steps, and timing the second step in order not to create undesired overhang at the clamping points. We do not report in the main text data pertaining to the device design in Fig. 3: the design was formulated when we were not fully aware of the buckling constraints outlined above, and exhibited pronounced static deformation in the largest branches. Correspondingly, the measured Q of $\sim 9\times10^6$ was more than an order of magnitude lower than the numerical dissipation dilution prediction. In a similar manner, we expect that a backside window may be included in the future with the device of Fig. 4a of the main text, although probably at the cost of a reduction in sample yield (e.g. due to more intense liquid flows during wet etching, handling and rinsing).

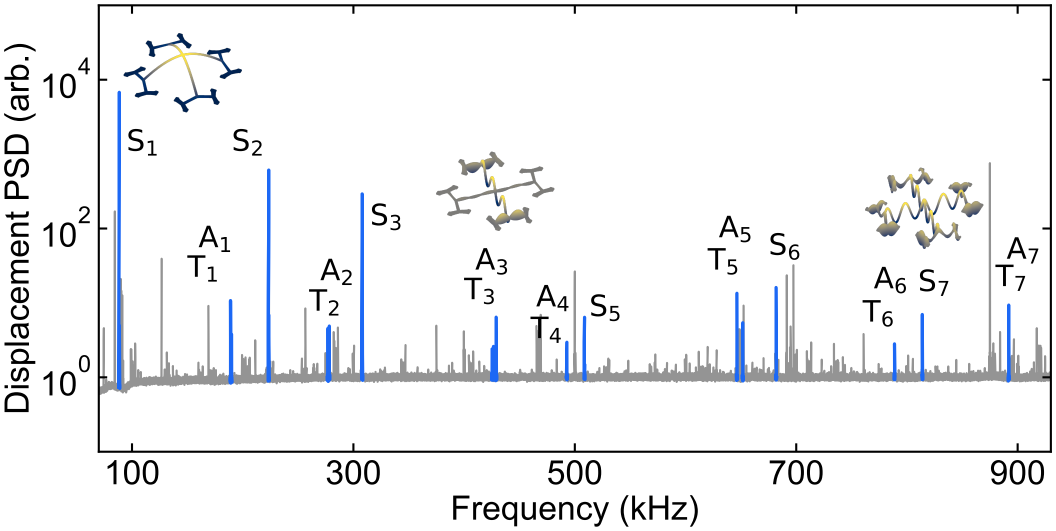

Figure 4: Thermomechanical noise spectrum of a trampoline with branching tethers. The gray line is the power spectral density of the homodyne record, where we highlight in blue the Brownian motion peaks of the trampoline resonator. The insets display the displacement patterns of modes S_1, T_3 and S_7.

4 Thermomechanical spectrum of a trampoline with branching tethers

In Fig. 4 we display the thermomechanical noise spectrum recorded by shining the probe laser on the pad of a trampoline membrane with branching tethers. Resonance peaks were distinguished from spurious noise peaks by comparison with a similar spectrum obtained by reflecting the probe off the chip surface, and are highlighted in blue. The trace is normalized to the level of shot noise.

The low-frequency spectrum of the trampoline with branching tethers presents flexural mode families similar to those of regular trampoline membranes. We adopt the notation of [9]: ‘S’ indicates

symmetric displacement patterns, with an antinode at the location of the pad, ‘T’, modes where one tether vibrates out of the plane, with a node at the pad, and the second undergoes torsion, and ‘A’, modes where the two tethers undergo flexural displacement with a π phase shift. ‘T’ modes always appear in degenerate pairs, and ‘A’ modes at a slightly higher frequency. The interferometer is most sensitive to out-of-plane motion, and purely torsional and in-plane flexural resonances could not be detected. Modes localized to high-generation segments appear at frequencies beyond the acquisition band.

5 Optical backaction from the measurement beam

The $780\,\mathrm{nm}$ optical probe that was used for the mechanical quality factor characterization in the room temperature setup could exert backaction on vibrational modes and affect the observed energy decay rates. This effect had to be avoided in order to extract intrinsic mechanical properties. The magnitude and the sign of the optically induced damping depended sensitively on the sample type and the position of the laser spot. In many cases, the optical damping was negligible, while sometimes it was strong enough to self-excite resonator modes under continuous illumination. We did not make a detailed physical model for the optical damping, but we conjecture that the mechanism behind it involved the standing wave formed due to the reflection of the probe from the surface of the chip underneath the resonator.

To eliminate the effects of optical damping, we gated the ringdown measurements, by keeping the sample illuminated by the probe light only during short intervals of time. The total duration

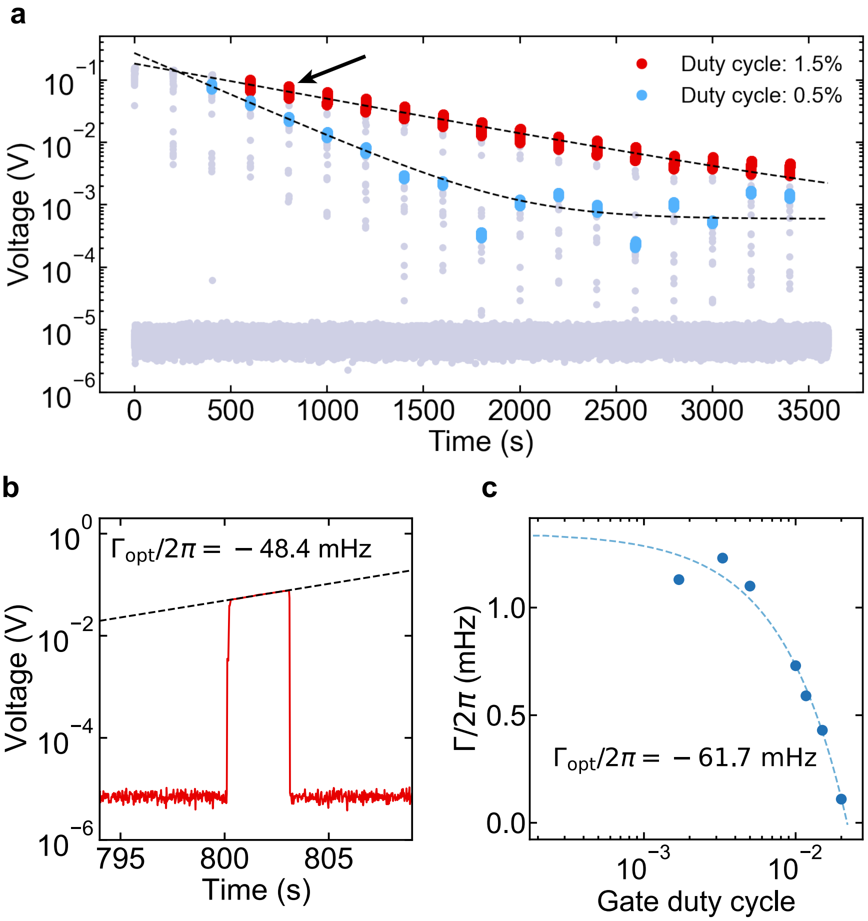

Figure 5: Optical antidamping of a trampoline resonator. **a,** Ringdown traces with different gate duration, exhibiting different optical antidamping rates. Gray points correspond to the noise background and the rising/falling edges of the gates, and are excluded from the fits. **b,** Homodyne signal within the gate indicated with a black arrow in panel a. The positive slope of the voltage within the gate implies antidamping. **c,** Extracted net damping rate as a function of gate duty cycle. A linear fit used to extract the optical antidamping rate yields $\Gamma_{\text{opt}} \approx 2\pi \times (-61.7\,\text{mHz})$ (in fair agreement with the growth of the displacement signal within a single gate, shown in panel b) and an intrinsic dissipation rate of $\Gamma_m \approx 2\pi \times 1.3\,\text{mHz}$.

of time over which the probe light was on relative to the entire measurement time was typically between 0.1% and 1% in our measurements. We refer to this ratio as duty cycle. For each resonance, the absence of optical backaction was confirmed by measurements with different duty cycles and/or laser beam positions on the sample.

An example of particularly strong optical backaction is illustrated in Fig. 5. Panel a shows two ringdown measurements of the fundamental mode of a trampoline resonator with branching tethers (the fundamental mode in the spectrum of Fig. 4) performed with the $780\,\mathrm{nm}$ probe beam directed at the resonator central pad. The displayed measurements made at two different gate duty cycles had decay rates differing of a factor of 2.5. A deviation from exponential decay manifests at high amplitudes, close to the trace beginning. This is due to the strong phase modulation imparted on the probe upon reflection from the trampoline, whose oscillation amplitude approaches the wavelength. The transduction of the displacement signal by the interferometer becomes nonlinear, and higher-order sidebands appear in the photocurrent spectrum [10]. In these traces, therefore, only the linear amplitude decay regime was fit, with a simple exponential model.

When the duty cycle was beyond $\approx 1.6\%$, the mode was self-excited, as shown by a close-up plot of one of the measurement intervals in Fig. 5b. The optical damping rate inferred from this data is $\Gamma_{\mathrm{opt}} = -2\pi \times 48.4\,\mathrm{mHz}$ at the continuous optical power of around $100\,\mu\mathrm{W}$. In gated measurements, the optical antidamping was reduced proportionally to the duty cycle, which is confirmed by the data in Fig. 5c. While Fig. 5 presents an extreme example of optical backaction, usually such effects could be reduced or eliminated entirely by directing the laser beam to a position

of the device where the mode amplitude was lower.

In our cryogenic setup, quality factor measurements on binary tree beams were performed using a $1550\,\mathrm{nm}$ gated probe laser, although no signatures of optical backaction were observed.

1. Unterreithmeier, Q. P., Faust, T. & Kotthaus, J. P. Damping of nanomechanical resonators. *Physical Review Letters* **105**, 027205 (2010).

2. Yu, P.-L., Purdy, T. P. & Regal, C. A. Control of material damping in high-Q membrane microresonators. *Physical Review Letters* **108**, 083603 (2012).

3. Villanueva, L. G. & Schmid, S. Evidence of surface loss as ubiquitous limiting damping mechanism in sin micro- and nanomechanical resonators. *Physical Review Letters* **113**, 227201 (2014).

4. Fedorov, S. A. *et al.* Generalized dissipation dilution in strained mechanical resonators. *Physical Review B* **99**, 054107 (2019).

5. Fedorov, S. A., Beccari, A., Engelsen, N. J. & Kippenberg, T. J. Fractal-like mechanical resonators with a soft-clamped fundamental mode. *Physical Review Letters* **124**, 025502 (2020).

6. Tsaturyan, Y., Barg, A., Polzik, E. S. & Schliesser, A. Ultracoherent nanomechanical resonators via soft clamping and dissipation dilution. *Nature Nanotechnology* **12**, 776–783 (2017).

7. González, G. I. & Saulson, P. R. Brownian motion of a mass suspended by an anelastic wire. *The Journal of the Acoustical Society of America* **96**, 207–212 (1994).

8. Ghadimi, A. H. *et al.* Elastic strain engineering for ultralow mechanical dissipation. *Science* **360**, 764–768 (2018).

9. Reinhardt, C., Müller, T., Bourassa, A. & Sankey, J. C. Ultralow-noise SiN trampoline resonators for sensing and optomechanics. *Physical Review X* **6**, 021001 (2016).

10. Bereyhi, M. *et al.* Nanomechanical resonators with ultra-high-Q perimeter modes. *arXiv preprint arXiv:2108.03615* (2021).
